# Assessing the impact of critical care training on pharmacy students in Egypt: a pre-post study

**DOI:** 10.1186/s12909-024-06427-6

**Published:** 2024-12-20

**Authors:** Hebatallah Ahmed Mohamed Moustafa, Alaa Essawy Hamid, Gehad Hassoub, Amira B. Kassem

**Affiliations:** 1 Clinical pharmacy and pharmacy practice department, Faculty of Pharmacy, Badr University in Cairo, Badr City, Egypt; 2 Pharmacy department, Zamzam Hospital, Alexandria, Egypt; 3 Department of Clinical Pharmacy and Pharmacy Practice, Faculty of Pharmacy, Damanhour University, Damanhour, Egypt

**Keywords:** Critical care, Training, Pharmacy students, Experiential learning, Pharmacy education

## Abstract

**Background:**

Transition to independent pharmacy practice is challenging. Undergraduate and postgraduate pharmacy students in low-to-middle-income countries (LMICs) currently receive minimal experiential learning in critical care.

**Objective:**

To assess the critical care training course’s impact on pharmacy students’ knowledge, self-efficacy, and self-esteem, as well as their satisfaction with the course.

**Method:**

In a pre-post interventional study, pharmacy students took a 10-day critical care training course with experiential learning. They completed a knowledge questionnaire covering various critical care topics, the 18 questions Pharmacy Self-efficacy and Self-esteem Study Questionnaire, and a satisfaction questionnaire. Data was analyzed using IBM SPSS version 20.0.

**Results:**

A total of 72 trainees participated in the study. The average score for pre-course self-efficacy and self-esteem significantly increased post-course, with a median (IQR) of 3.75 (3.50–3.94) compared to 3.56 (3.28–3.78) pre-course, with a *p*-value of less than 0.05. Furthermore, their overall knowledge score significantly increased from baseline to post-course, with the median (IQR) rising from 0.53 (0.50–0.61) to 0.98 (0.97–1.0), with a *p*-value of less than 0.05. Their course satisfaction average total score was 45.08 (SD ± 5.41) (on a scale of 10–50).

**Conclusion:**

The present study provides clear evidence that integrating this critical care training course into a structured curriculum for pharmacy students using experiential learning can act as a facilitator of knowledge enrichment, increase their self-esteem and self-efficacy, and make them satisfied with learning. Larger long duration studies are needed to support this evidence.

**Supplementary Information:**

The online version contains supplementary material available at 10.1186/s12909-024-06427-6.

## Introduction

Critical care medicine is a multidisciplinary healthcare system that provides care for patients who are suffering from acute life-threatening conditions [1]. The critical care unit is a unique area of a hospital that is specially designed and equipped to deal with such cases with aggressive therapy to improve patient morbidity and mortality [2]. This can be successfully achieved through sufficient education, training, and collaboration among all critical care team members, including physicians, nurses, respiratory therapists, critical care pharmacists, and others [3].

Despite the difficulty of conducting research in the critical care setting, the pharmacy profession’s added value in critical care cannot be overlooked [4, 5]. Critical care pharmacists can provide a broad range of services, such as medication order verification, pharmacological ventilator-weaning protocol development, medication reconciliation, therapeutic drug monitoring, adjusting doses for narrow therapeutic index drugs, and conducting research [5–8]. They actively engage in the daily multidisciplinary team (MDT) round and communicate with other team members to optimize therapeutic plans [9, 10]. Although pharmacists’ involvement in critical care can lead to better outcomes and lower costs [11–14], many critical care units lack the presence of qualified pharmacists with the necessary therapeutic skills [15, 16]. Lack of critical care exposure and experience can negatively affect the students’ confidence and self-efficacy [17, 18].

Egypt’s pharmaceutical education is undergoing a transition from the traditional 5-year bachelor’s degree (B.Pharm) to the new doctor of pharmacy (Pharm D) and doctor of pharmacy clinical (Pharm D clinical) degrees. This transition includes an additional internship year, during which students will rotate through various sectors of industry, quality control, hospital setting, etc. The rotation sites will vary based on the student’s preference and the training sites provided by their faculty. Notably, even with the addition of an experiential learning internship year, set to commence in the fall semester of the 2024–2025 academic year, not all students will have the opportunity to undergo training in an ICU during their final year [19].

The positive role of pharmacists in improving clinical outcomes, improving quality of life, and reducing health service use in low-to-middle-income countries (LMICs) was previously reported [20, 21]. Therefore, there is a great need for specialized pharmacists in LMICs such as Egypt, especially critical care pharmacists [21, 22]. Because critical care pharmacists working in LMICs need certain qualifications to face the unique challenges needed in less-resourced settings [23, 24], it is necessary to reform pharmacy education and practice models to ensure graduates’ competence and optimize resource utilization [22, 25, 26].

Critical care practice is not mandated per se in most of the undergraduate curricula of pharmacy educational programs, and not all pharmacy students, including Egyptian pharmacy students, get the chance to be exposed to experiential training inside the critical care unit during their classic didactic learning journey [1, 5, 19]. These courses are mostly provided as supplemental or elective courses [27, 28]. Moreover, many pharmacy students find critical care topics difficult and clinical rotations difficult to pass, which further contributes to the low number of critical care experiences among pharmacists [1]. Some critical care pharmacists never receive training in critical care settings other than the standard courses in the faculty curriculum [5].

To be proactive and make the most of their role in the practice [29], pharmacy students need at least the basic knowledge, skills, and confidence to become prepared to deal with the complexity of the critical care practice. Although students “learn by doing” and experiential learning [30], there are deficiencies in effective bedside teaching, applying competency-based education, growing consultation skills, and ongoing regular assessment of professional activities in healthcare education [29, 31–33]. Practice allows for the acquisition of this through trial and error. Otherwise, it should be provided by specialized residency or by completing a well-established training program [34, 35]. Pharmacist trainees’ communication skills can improve during experiential learning as they learn how to speak to a real person and develop cultural sensitivity [36].

Based on our knowledge and literature review, this is the first prospective study to assess the impact of training in a critical care unit on pharmacy students’ self-efficacy, self-esteem, and knowledge. Most of the published work is concerned with nursing and medical students [31, 37], instead of pharmacy students. There is a paucity of literature describing providing training for pharmacy students in the critical care setting globally and in LMICs, as most previous studies were conducted in developed countries and didn’t include critical care on-site training [28]. The current study aims to evaluate the trainees’ knowledge on the topics covered in the training course, quantify the changes in their self-esteem and self-efficacy over the 10-day course duration, and assess the participant’s satisfaction with the training course.

## Methods

### Study design

This is an interventional pre-post study that was conducted over 3 months in the form of 8 waves, each of which lasted for 10 days. All students electively registered for the elective summer training course were included in the study after being asked if they had any previous exposure to the critical care unit. The hospital where the training took place provided the trainees with professional clinical pharmacy preceptors specializing in critical care, along with their academic supervisor (HAAM). All questionnaires were administered to the trainees as hard copies. At baseline, trainees filled out a questionnaire on sociodemographic data. Daily pre- and post-tests were given to evaluate students’ knowledge about the daily topic. Students took the pretest before attending the day’s lectures, daily rounds, and case discussions, and answered the post-test at the end of the day, identical to the pre-test. Their answers were assessed in comparison with the correct answer written in bold font (Tables 1, 2, 3, 4, 5, 6, 7, 8, 9, 10 and 11S). Marks were deducted for incorrect answers. We calculated the sum of the correct answers to the knowledge domain questions for each topic, both before and after the training program.

We invited all the students to participate in the self-esteem and self-efficacy questionnaire (Table 12S) on day 1, prior to the start of the training course, and again 10 days later, at the end of the training course. We computed the mean of the trainees’ reported numbers to derive a measure of their self-efficacy, self-esteem, and overall self-efficacy and self-esteem.

The assessment of the student’s course satisfaction was conducted only on day 10, at the end of the training course. We calculated the average total score for course satisfaction (Table 13S). Before the study commenced, a pilot test of the entire training program was conducted on three practicing pharmacy students to ensure its appropriateness.

### Participants

A training course was electively offered independently to pharmacy students from four different Egyptian universities: Alexandria, Pharos, the Arab Academy for Science, Technology, and Maritime Transport, and 6 October University. The study participants included undergraduate 4th-year B.Pharm pharmacy students (from the old Egyptian pharmaceutical education program) and post-graduate professional master’s degrees (providing theoretical and experiential clinical training over 2 years). Neither B.Pharm nor professional master students were exposed to experiential learning in the critical care setting in the curricula before this training experience.

### Setting

The study took place at a small (80 beds, of which 20 are critical care unit beds) general private non-academic hospital in Alexandria, Egypt.

### Training course

The condensed training program was designed to offer opportunities for pharmacy students to grow the foundational knowledge, self-efficacy, and self-esteem necessary to provide care to critically ill patients. It was repeated in waves of six or seven trainees. The author, AEH, designed the training course based on the relevant literature on the topic. The students learned ten different topics over 10 different days, which included (a) phenytoin, (b) stroke, (c) pain and agitation, (d) delirium, (e) heart failure, (f) atrial fibrillation, (g) chronic kidney disease, (h) electrolyte disturbance, (i) evidence-based medicine (EBM), (j) sepsis, and k) pneumonia.

Every day of the 10-day wave began with a one-hour lecture, followed by five hours of exposure to various experiential learning techniques. These techniques included participating in ward and critical care rounds with a multidisciplinary team, exploring case-based scenarios, interpreting lab results, dosing, mitigating medication errors and drug interactions, providing possible recommendations, making follow-up plans, and understanding the role of informed decision-making, empathy, and patient autonomy. The training program allowed for the gradual development of students’ competence and capabilities as the preceptors encouraged trainees to play an active role, starting with merely observing and then performing small tasks under their supervision.

The training program was designed by a professional clinical pharmacist (AEH) and was supervised by one academic member of the clinical pharmacy and pharmacy practice department (HAAM) in the faculty of pharmacy. Collaboration between staff members and healthcare-system-based practitioners in critical care was previously recommended [5].

### Instruments

The students were asked to fill in a section covering their demographic information (age, gender, B-pharm or professional-masters, public or private university, “grade point average” (GPA)), in addition to questionnaires measuring knowledge, self-efficacy, self-esteem, and course satisfaction (Supplementary File).

#### Knowledge assessment tests

Under the supervision of one of the authors (the clinical pharmacy department head, AEH) and after reviewing the relevant literature, the researchers developed a set of questions to assess the student’s knowledge level of eleven critical care practice topics (Tables 1, 2, 3, 4, 5, 6, 7, 8, 9, 10 and 11S). True/false and multiple-choice questions are included in the closed-ended structure of the questionnaire.

The questionnaire’s content validity was verified by a panel of five experts in clinical pharmacy and critical care using the content validity index (CVI). On a 4-point scale from “not relevant” to “highly relevant,” the reviewers ranked each item’s relevancy [38]. An acceptable level of validity was indicated by Scale-CVI of 0.82 [39]. Two independent clinical pharmacists conducted a pilot study on three pharmacy students to assess the suitability and clarity of the knowledge instrument.

Internal consistency was measured using Cronbach’s alpha with a cut-off point of 0.7 or higher [40]. After analyzing the initial Cronbach’s alpha, specific questions were discarded to improve the questionnaire’s reliability. Cronbach’s alpha was calculated for the 58 items’ final version, yielding a value of 0.73, indicating satisfactory overall reliability.

#### Self-efficacy and self-esteem questionnaires

The Pharmacy Self-efficacy and Self-esteem Study Questionnaire was used after obtaining permission from the author [41]. The first eight questions assess self-efficacy with a possible score (ranging from 8 to 40), and the subsequent ten questions assess self-esteem using a 5-point Likert scale ranging from strongly disagree to strongly agree, with a possible score ranging from 10 to 50 (Table 12S).

#### Course-satisfaction questionnaire

a 10-item survey evaluating students’ satisfaction was derived from a previous study [42], with minor modifications. Responses were recorded on a Likert scale with 1 indicating “completely disagree” and 5 indicating “completely agree” (Table 13S). The same procedures for assessing reliability and validity in Knowledge assessment tests were employed. The results revealed acceptable reliability and content validity (Cronbach’s α = 0.7 and Scale-CVI = 0.8).

### Sample size calculation

The sample size was computed based on the reported mean difference in pharmacy students’ knowledge in earlier research assessing the benefits of practical training [43]. The study revealed an improvement in the mean of the knowledge from 6.17 ± 0.76 in pretest to 6.63 ± 0.58. Using G*Power software, the minimum required sample size was 31 participants, based on a power of 90%, a significance level of 0.05, and 20% compensation for loss to follow-up.

### Statistical analysis of the data

The data was entered into the computer and statistically analyzed using the IBM SPSS software (version 20.0). (Armonk, NY: IBM Corp.). Qualitative data were presented as numbers and percentages. The normality of the distribution was tested by the Kolmogorov-Smirnov test. Quantitative data were described using either range (minimum and maximum), mean, standard deviation, median, or interquartile range (IQR). The significance of the results was set at the 5% level. To compare between two groups, the Mann-Whitney test was used for abnormally distributed quantitative variables. We used the Kruskal-Wallis test for abnormally distributed quantitative variables to compare more than two studied groups. For abnormally distributed quantitative variables, the Wilcoxon signed ranks test was used to compare two periods. To correlate between two quantitative variables distributed abnormally, we used the Spearman coefficient.

## Results

The training was held at a private hospital in Alexandria. Seventy-two pharmacy students from four Egyptian universities participated in the training, of which 56 students (77.8%) were females, 22 students (30.6%) were public university students, and 5 students (6.9%) were post-graduate (professional masters) students. The mean age was 22.26 (SD ± 1.84) and the mean GPA was 3.02 (SD ± 0.55) (Table 1).

**Table 1:** Distribution of the studied cases according to demographic data (*n* = 72)

	No.	%
Gender		
Male	16	22.2
Female	56	77.8
Age		
<22	19	26.4
22–24	48	66.7
≥25	5	6.9
Min. – Max.	19.0–32.0	
Mean ± SD.	22.26 ± 1.84	
Median (IQR)	22.0 (21.0–22.50)	
University		
PUA	42	58.3
6 October	6	8.3
Alexandria	22	30.6
AAST	2	2.8
University type		
Public	22	30.6
Private	50	69.4
GPA		
<2	3	4.2
2–2.49	11	15.3
2.5–3	15	20.8
>3	43	59.7
Min. – Max.	1.91–4.0	
Mean ± SD.	3.02 ± 0.55	
Median (IQR)	3.16 (2.68–3.40)	
Education		
Undergraduate	67	93.1
Postgraduates	5	6.9

IQR: Inter quartile range SD: Standard deviation

PUA: Pharos University in Alexandria

AAST: Arab academy for science and technology

GPA: Grade Point Average

**Fig. 1:**
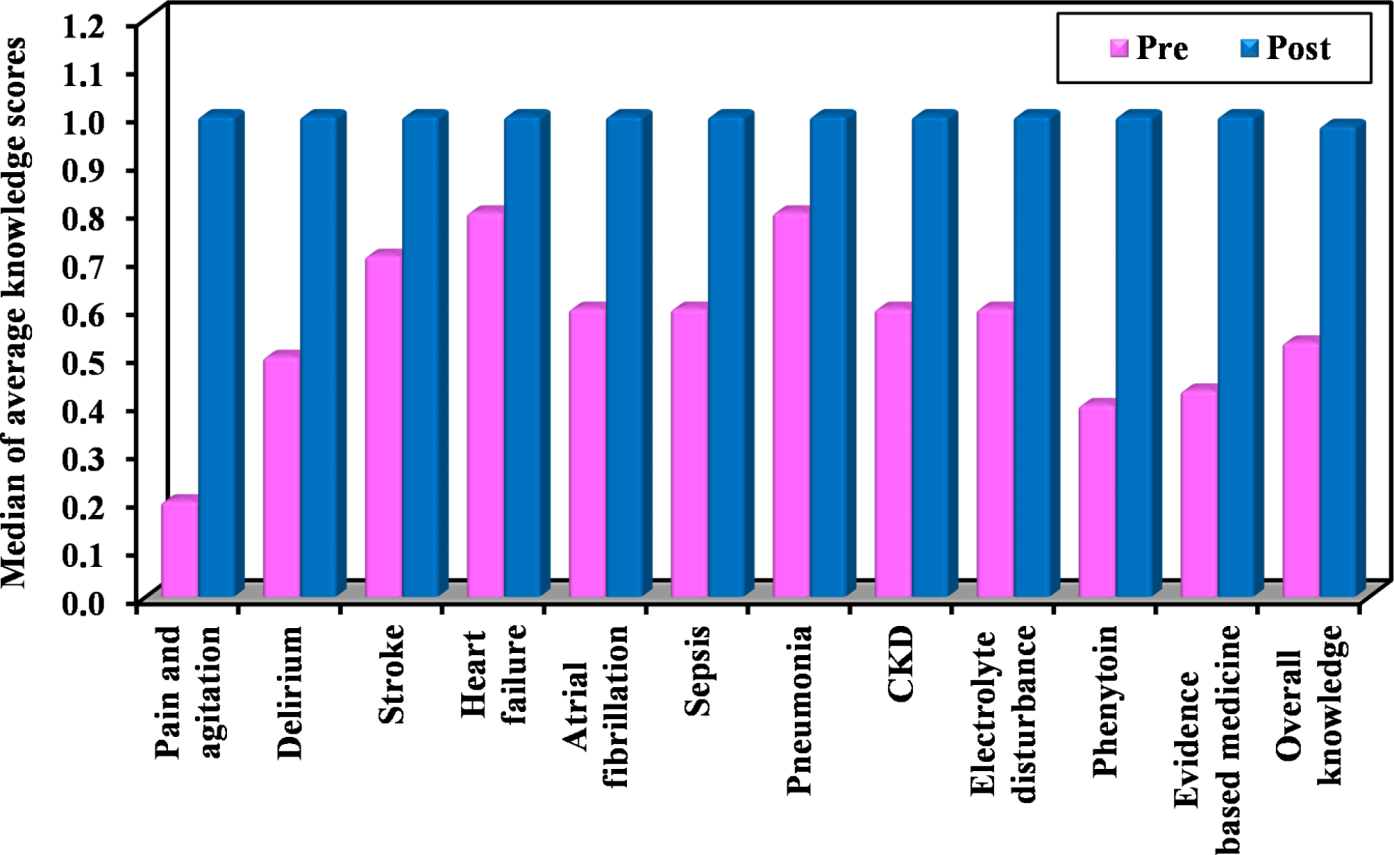
Comparison between pre- and post-training knowledge scores CKD: Chronic kidney disease

All topics exhibited a statistically significant rise in students’ scores over the training course duration (*p* ≤ 0.05) (Fig. 1). The top 5 topics that showed the greatest increase in the total mean scores were: (a) pain and agitation; (b) phenytoin; (c) EBM; (d) delirium and chronic kidney disease (Table 14S, 15S, 16S, and 17S). The average overall knowledge score showed a significant increase from baseline to post-course (*p* < 0.05) (Table 2).

**Table 2:** Comparison between pre and post according to knowledge scores (*n* = 72)

	Pre	Post	Difference	Rank	*p*
Pain and agitation	0.36 ± 0.25	0.98 ± 0.08	0.62 ± 0.27	**1**	< 0.001^*^
Delirium	0.44 ± 0.24	0.95 ± 0.11	0.51 ± 0.25	**4**	< 0.001^*^
Stroke	0.73 ± 0.15	0.98 ± 0.05	0.26 ± 0.15	**10**	< 0.001^*^
Heart failure	0.76 ± 0.19	0.99 ± 0.03	0.23 ± 0.19	**11**	< 0.001^*^
Atrial fibrillation	0.61 ± 0.24	1.0 ± 0.02	0.39 ± 0.24	**7**	< 0.001^*^
Sepsis	0.62 ± 0.23	0.97 ± 0.08	0.36 ± 0.24	**8**	< 0.001^*^
Pneumonia	0.68 ± 0.23	1.0 ± 0.02	0.31 ± 0.24	**9**	< 0.001^*^
CKD	0.52 ± 0.26	0.96 ± 0.09	0.44 ± 0.27	**5**	< 0.001^*^
Electrolyte disturbance	0.55 ± 0.23	0.96 ± 0.11	0.41 ± 0.25	**6**	< 0.001^*^
Phenytoin	0.39 ± 0.27	1.0 ± 0.02	0.61 ± 0.27	**2**	< 0.001^*^
Evidence based	0.40 ± 0.17	0.96 ± 0.09	0.56 ± 0.18	**3**	< 0.001^*^
Overall knowledge	**0.55 ± 0.08**	**0.98 ± 0.02**	**0.42 ± 0.08**		< 0.001^*^

SD: Standard deviation

Z: Wilcoxon signed ranks test

p: *p* value for comparing between pre and post

*: Statistically significant at *p* ≤ 0.05

**Fig. 2:**
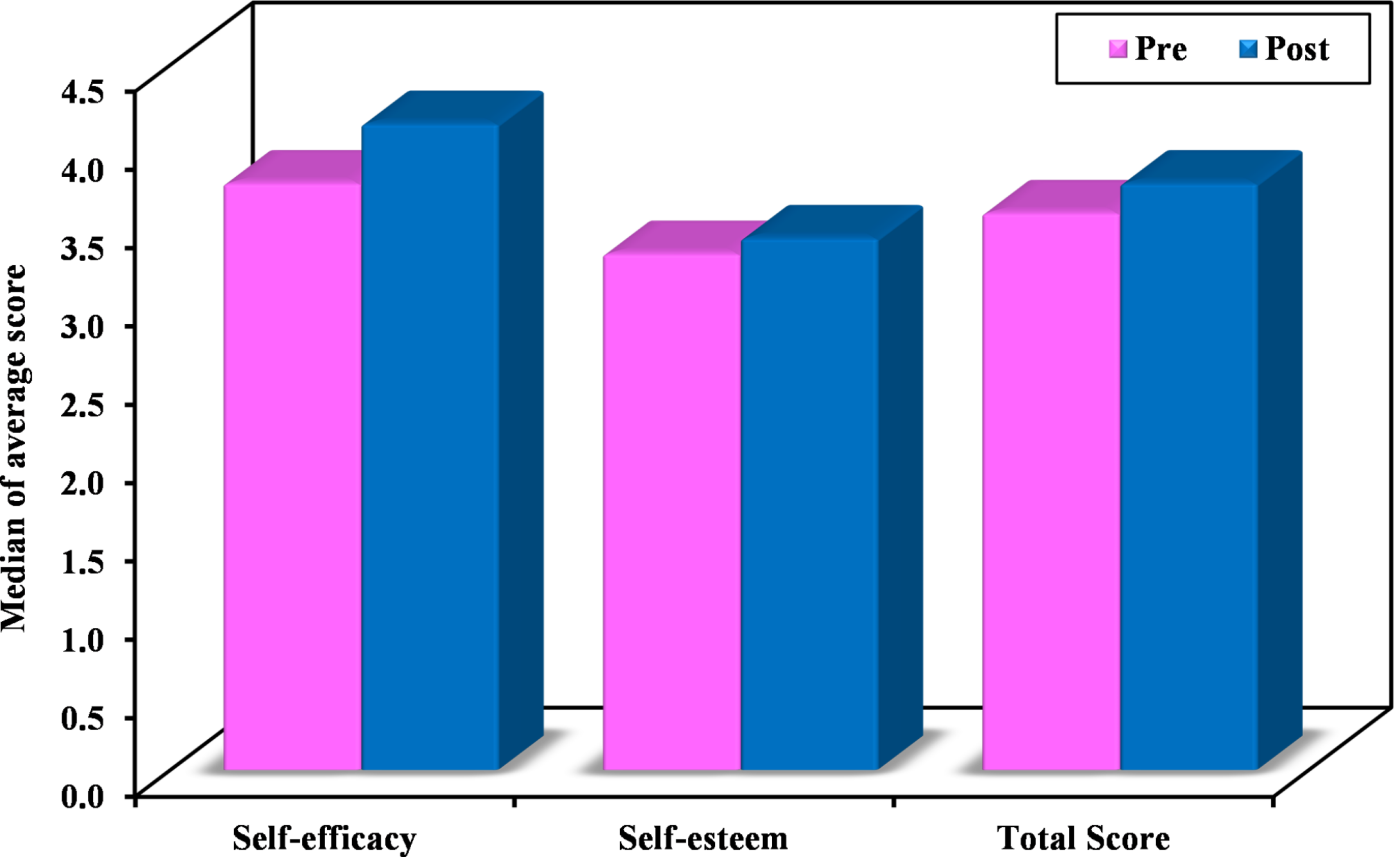
Comparison between pre- and post-training self-efficacy, self-esteem, and total scores of self-efficacy and self-esteem

Following the training course, the average self-efficacy students’ scores improved significantly (*p* < 0.05) (Table 3). There was also a significant increase in the overall average mean score of both self-efficacy and self-esteem after finishing the training course (*p* < 0.05). On the other hand, self-esteem scores displayed a notable increase, though this change was not statistically significant (Fig. 2).

**Table 3:** Comparison between pre and post according to self-efficacy and self-esteem (*n* = 72)

	Pre	Post	*p*
Self-efficacy			
Average Score			< 0.001^*^
Min. – Max	1.0–5.0	1.0–5.0	
Median (IQR)	3.75 (3.31–4.19)	4.13 (3.75–4.63)	
Self-esteem			
Average Score			0.203
Min. – Max	1.0–4.10	1.0–5.0	
Median (IQR)	3.30 (3.05–3.70)	3.40 (3.10–3.70)	
Total Score			
Average Score			0.001^*^
Min. – Max	1.0–4.44	1.0–5.0	
Median (IQR)	3.56 (3.28–3.78)	3.75 (3.50–3.94)	

IQR: Inter quartile range SD: Standard deviation

Z: Wilcoxon signed ranks test

p: *p* value for comparing between pre and post *: Statistically significant at *p* ≤ 0.05

Table 4 represents the level of course satisfaction reported by the students after training attendance. Overall, the training program was well received by the trainees. The course satisfaction total average score was 45.08 (SD ± 5.41) (on a scale of 10–50).

**Table 4:** Distribution of the studied cases according to course satisfaction tool (*n* = 72)

Q	Course satisfaction tool	Strongly disagree		Disagree		Can’t decide		Agree		Strongly agree	
		No.	%	No.	%	No.	%	No.	%	No.	%
1	Overall, I enjoyed the course	1	1.4	0	0.0	0	0.0	12	16.7	59	81.9
2	Overall, the course improved my knowledge of critical care	1	1.4	0	0.0	1	1.4	15	20.8	55	76.4
3	Overall, the course gave me the experience/skills I wanted or needed	1	1.4	0	0.0	2	2.8	36	50.0	33	45.8
4	Overall, the course met my learning needs	1	1.4	0	0.0	3	4.2	33	45.8	35	48.6
5	Overall, the learning experience was better than expected	1	1.4	2	2.8	2	2.8	19	26.4	48	66.7
6	Overall, the content of the course was easy to follow	1	1.4	1	1.4	5	6.9	37	51.4	28	38.9
7	Overall, I am satisfied with the pace of the course	1	1.4	1	1.4	4	5.6	33	45.8	33	45.8
8	Overall, I am satisfied with the way the course was delivered	1	1.4	0	0.0	4	5.6	2	37.5	40	55.6
9	Overall, the skills and knowledge acquired during the course will help me in my job	3	4.2	0	0.0	1	1.4	21	29.2	47	65.3
10	Overall, the course is very useful to my students,	1	1.4	0	0.0	1	1.4	15	20.8	55	76.4
	**Total Score**	**(10–50)**									
	Min. – Max	10.0–50.0									
	Mean ± SD.	45.08 ± 5.41									
	Median (IQR)	46.50 (43.0–48.0)									

Table 5 illustrates the correlation between self-efficacy, self-esteem, overall self-efficacy and self-esteem, and demographic data. Both self-efficacy and overall self-efficacy and self-esteem were significantly higher in public university students pre-training compared to private university students and in students with higher GPAs compared with those with lower GPAs (*p* < 0.05). Students from public universities had a greater difference in overall self-efficacy and self-esteem scores between pre- and post-training. Also, those with the lowest GPAs had the most significant increase in their self-efficacy scores post-training versus pre-training (*p* < 0.05) (Table 6).

**Table 5:** Relation between self-efficacy, self-esteem and overall with demographic data

	Self-efficacy				Self-esteem				Overall			
	Pre		Post		Pre		Post		Pre		Post	
	Mean ± SD.	Median	Mean ± SD.	Median	Mean ± SD.	Median	Mean ± SD.	Median	Mean ± SD.	Median	Mean ± SD.	Median
Gender												
Male	3.66 ± 0.56	3.75	4.07 ± 0.72	3.94	3.28 ± 0.46	3.20	3.84 ± 0.79	3.80	3.45 ± 0.41	3.53	3.94 ± 0.61	3.72
Female	3.80 ± 0.73	3.75	4.19 ± 0.63	4.13	3.31 ± 0.50	3.30	3.36 ± 0.55	3.35	3.53 ± 0.50	3.56	3.73 ± 0.52	3.75
U(p)	**383.500 (0.381)**		**395.000 (0.471)**		**403.000 (0.541)**		**318.000 (0.077)**		**392.000 (0.447)**		**410.500 (0.611)**	
Age												
< 22	3.89 ± 0.66	3.75	4.39 ± 0.44	4.38	3.38 ± 0.34	3.40	3.45 ± 0.55	3.40	3.61 ± 0.42	3.56	3.87 ± 0.41	3.72
22–24	3.72 ± 0.71	3.75	4.06 ± 0.70	4.13	3.27 ± 0.54	3.20	3.45 ± 0.66	3.40	3.47 ± 0.49	3.50	3.72 ± 0.58	3.78
≥ 25	3.80 ± 0.81	4.13	4.35 ± 0.61	4.13	3.38 ± 0.49	3.20	3.66 ± 0.79	3.50	3.57 ± 0.58	3.83	3.97 ± 0.64	3.67
H(p)	**0.627 (0.731)**		**4.308 (0.116)**		**0.665 (0.717)**		**0.385 (0.825)**		**1.174 (0.556)**		**1.134 (0.567)**	
University												
PUA	3.54 ± 0.72	3.50	4.16 ± 0.69	4.13	3.23 ± 0.53	3.20	3.47 ± 0.71	3.35	3.37 ± 0.53	3.44	3.78 ± 0.65	3.75
6 October	4.19 ± 0.66	4.25	3.92 ± 0.98	4.13	3.62 ± 0.37	3.70	3.82 ± 0.55	3.85	3.87 ± 0.32	3.78	3.86 ± 0.34	3.78
Alexandria	4.13 ± 0.49	4.13	4.26 ± 0.46	4.13	3.38 ± 0.40	3.35	3.37 ± 0.53	3.40	3.71 ± 0.26	3.67	3.77 ± 0.37	3.69
AAST	3.44 ± 0.09	3.44	3.94 ± 0.62	3.94	3.15 ± 0.07	3.15	3.35 ± 0.21	3.35	3.28 ± 0.08	3.28	3.61 ± 0.39	3.61
H (p)	**14.625** ^*^ **(0.002** ^*^ **)**		**0.993 (0.803)**		**5.080 (0.166)**		**3.479 (0.324)**		**16.026** ^*^ **(0.001** ^*^ **)**		**0.783 (0.854)**	
University type												
Public	4.13 ± 0.49	4.13	4.26 ± 0.46	4.13	3.38 ± 0.40	3.35	3.37 ± 0.53	3.40	3.71 ± 0.26	3.67	3.77 ± 0.37	3.69
Private	3.61 ± 0.72	3.50	4.12 ± 0.71	4.13	3.27 ± 0.52	3.20	3.51 ± 0.68	3.40	3.42 ± 0.52	3.47	3.78 ± 0.61	3.78
U(p)	**295.000** ^*^ **(0.002** ^*^ **)**		**502.000 (0.555)**		**476.500 (0.367)**		**459.000 (0.264)**		**331.000** ^*^ **(0.007** ^*^ **)**		**519.000 (0.704)**	
GPA												
< 2	2.50 ± 1.30	3.25	4.25 ± 0.13	4.25	2.50 ± 1.41	2.70	3.23 ± 0.42	3.10	2.50 ± 1.33	2.94	3.69 ± 0.22	3.56
2–2.49	3.70 ± 0.77	3.75	4.45 ± 0.52	4.38	3.24 ± 0.44	3.20	3.43 ± 0.59	3.40	3.44 ± 0.54	3.56	3.88 ± 0.45	3.78
2.5–3	3.44 ± 0.52	3.38	4.10 ± 0.76	4.00	3.37 ± 0.44	3.20	3.80 ± 0.78	3.70	3.40 ± 0.31	3.44	3.93 ± 0.63	3.78
> 3	3.99 ± 0.55	3.88	4.11 ± 0.65	4.13	3.36 ± 0.38	3.30	3.37 ± 0.58	3.40	3.64 ± 0.32	3.61	3.70 ± 0.54	3.72
H (p)	**14.447** ^*^ **(0.002** ^*^ **)**		**3.132 (0.372)**		**2.419 (0.490)**		**2.897 (0.408)**		**8.296** ^*^ **(0.040** ^*^ **)**		**0.861 (0.835)**	
Education												
Undergraduates	3.73 ± 0.70	3.75	4.15 ± 0.66	4.13	3.30 ± 0.48	3.30	3.49 ± 0.65	3.40	3.49 ± 0.48	3.56	3.78 ± 0.55	3.78
Postgraduates	4.23 ± 0.60	4.38	4.30 ± 0.43	4.13	3.36 ± 0.58	3.30	3.16 ± 0.38	3.20	3.74 ± 0.35	3.83	3.67 ± 0.36	3.50
U(p)	**96.00(0.118)**		**143.000 (0.605)**		**146.500 (0.651)**		**115.000 (0.258)**		**110.500 (0.214)**		**135.500 (0.490)**	

U: Mann Whitney test H: H for Kruskal Wallis test

p: *p* value for comparison between the studied categories

PUA: Pharos University in Alexandria

AAST: Arab academy for science and technology

GPA: Grade Point Average

*: Statistically significant at *p* ≤ 0.05

**Table 6:** Relation between (diff) self-efficacy, self-esteem, and overall with demographic data

	Self-efficacy(diff)		Self-esteem(diff)		Overall(diff)	
	Mean ± SD.	Median	Mean ± SD.	Median	Mean ± SD.	Median
Gender						
Male	0.41 ± 0.85	0.25	0.57 ± 0.88	0.60	0.50 ± 0.76	0.45
Female	0.40 ± 0.91	0.37	0.04 ± 0.77	0.00	0.20 ± 0.72	0.09
U(p)	**428.000 (0.786)**		**286.000** ^*^ **(0.028** ^*^ **)**		**362.000 (0.244)**	
Age						
< 22	0.50 ± 0.70	0.25	0.07 ± 0.63	-0.10	0.26 ± 0.54	0.11
22–24	0.34 ± 0.96	0.37	0.18 ± 0.86	0.00	0.25 ± 0.78	0.22
≥ 25	0.55 ± 1.02	0.62	0.28 ± 1.13	0.00	0.40 ± 1.02	0.06
H(p)	**0.628 (0.731)**		**0.682 (0.711)**		**0.068 (0.967)**	
University						
PUA	0.63 ± 1.01	0.75	0.24 ± 0.94	0.05	0.41 ± 0.88	0.28
6 October	-0.27 ± 0.45	-0.13	0.20 ± 0.40	0.15	-0.01 ± 0.22	0.01
Alexandria	0.14 ± 0.58	0.00	-0.01 ± 0.66	-0.05	0.06 ± 0.45	-0.06
AAST	0.50 ± 0.71	0.50	0.20 ± 0.28	0.20	0.34 ± 0.47	0.34
H (p)	**11.793** ^*^ **(0.008** ^*^ **)**		**2.323 (0.508)**		**7.915** ^*^ **(0.048** ^*^ **)**	
University type						
Public	0.14 ± 0.58	0.00	-0.01 ± 0.66	-0.05	0.06 ± 0.45	-0.06
Private	0.51 ± 0.98	0.56	0.23 ± 0.87	0.05	0.36 ± 0.82	0.28
U(p)	**391.000 (0.051)**		**430.500 (0.143)**		**363.500** ^*^ **(0.023** ^*^ **)**	
GPA						
< 2	1.75 ± 1.30	1.13	0.73 ± 1.76	0.20	1.19 ± 1.55	0.62
2–2.49	0.75 ± 0.77	0.75	0.19 ± 0.81	0.20	0.44 ± 0.73	0.17
2.5–3	0.66 ± 0.88	0.62	0.43 ± 0.99	0.40	0.53 ± 0.75	0.33
> 3	0.12 ± 0.78	0.00	0.02 ± 0.65	0.00	0.06 ± 0.59	0.06
H (p)	**12.007** ^*^ **(0.007** ^*^ **)**		**2.954 (0.399)**		**6.658 (0.084)**	
University type						
Undergraduates	0.42 ± 0.90	0.37	0.19 ± 0.83	0.00	0.29 ± 0.75	0.22
Postgraduates	0.08 ± 0.80	0.00	-0.20 ± 0.45	0.00	-0.08 ± 0.43	-0.22
U(p)	**126.0 (0.375)**		**121.0 (0.318)**		**94.50 (0.107)**	

U: Mann Whitney test H: H for Kruskal Wallis test

PUA: University in Alexandria

AAST: Arab academy for science and technology

GPA: Grade Point Average

p: *p* value for comparison between the studied categories

*: Statistically significant at *p* ≤ 0.05

Table 7 shows that overall knowledge at the baseline was significantly higher in students at public universities compared to students at private universities. Moreover, the difference between pre-and post-training overall knowledge was significantly higher among private university students versus those in public universities (*p* < 0.05).

**Table 7:** Relation between overall knowledge with demographic data

	Overall Knowledge					
	Pre		Post		Diff.	
	Mean ± SD.	Median	Mean ± SD.	Median	Mean ± SD.	Median
Gender						
Male	0.55 ± 0.09	0.56	0.97 ± 0.02	0.97	0.34 ± 0.19	0.36
Female	0.55 ± 0.08	0.53	0.98 ± 0.02	0.98	0.37 ± 0.16	0.43
U(p)	**431.500 (0.823)**		**336.500 (0.118)**		**378.500 (0.345)**	
Age						
< 22	0.53 ± 0.06	0.52	0.97 ± 0.02	0.98	0.37 ± 0.17	0.45
22–24	0.55 ± 0.08	0.53	0.98 ± 0.02	0.98	0.38 ± 0.16	0.42
≥ 25	0.63 ± 0.09	0.62	0.99 ± 0.01	0.98	0.20 ± 0.20	0.20
H(p)	**5.345 (0.069)**		**2.605 (0.272)**		**4.069 (0.131)**	
University						
PUA	0.53 ± 0.08	0.53	0.98 ± 0.02	0.98	0.41 ± 0.14	0.44
6 October	0.58 ± 0.06	0.58	0.98 ± 0.02	0.97	0.26 ± 0.21	0.33
Alexandria	0.59 ± 0.08	0.59	0.98 ± 0.02	0.98	0.30 ± 0.18	0.36
AAST	0.48 ± 0.00	0.48	0.97 ± 0.02	0.97	0.49 ± 0.02	0.49
H (p)	**10.703** ^*^ **(0.013** ^*^ **)**		**2.274 (0.517)**		**13.058** ^*^ **(0.005** ^*^ **)**	
University type						
Public	0.59 ± 0.08	0.59	0.98 ± 0.02	0.98	0.30 ± 0.18	0.36
Private	0.54 ± 0.08	0.53	0.98 ± 0.02	0.98	0.40 ± 0.15	0.44
U(p)	**344.00** ^*^ **(0.012** ^*^ **)**		**481.500 (0.386)**		**332.00** ^*^ **(0.008** ^*^ **)**	
GPA						
< 2	0.50 ± 0.03	0.48	0.95 ± 0.03	0.95	0.46 ± 0.04	0.45
2–2.49	0.58 ± 0.08	0.57	0.98 ± 0.02	0.98	0.28 ± 0.20	0.37
2.5–3	0.51 ± 0.10	0.48	0.97 ± 0.03	0.98	0.42 ± 0.16	0.47
> 3	0.56 ± 0.07	0.53	0.98 ± 0.02	0.98	0.36 ± 0.16	0.42
H (p)	**6.657 (0.084)**		**4.455 (0.216)**		**5.540 (0.136)**	
University type						
Undergraduates	0.55 ± 0.08	0.53	0.98 ± 0.02	0.98	0.38 ± 0.16	0.43
Postgraduates	0.62 ± 0.09	0.60	0.99 ± 0.01	0.98	0.21 ± 0.21	0.20
U(p)	**89.0 (0.084)**		**128.50 (0.399)**		**99.50 (0.135)**	

U: Mann Whitney test H: H for Kruskal Wallis test

PUA: Pharos University in Alexandria

AAST: Arab academy for science and technology

GPA: Grade Point Average

p: *p* value for comparison between the studied categories

*: Statistically significant at *p* ≤ 0.05

No differences were observed in satisfaction according to the demographic information (Table 8). The student’s overall knowledge before training was significantly positively correlated with both self-efficacy and overall self-efficacy and self-esteem, with a *p*-value of less than 0.05 (Table 9). In addition, there was a significant positive relationship between self-efficacy post-training and course satisfaction (*p* < 0.05) (Table 10).

**Table 8:** Relation between satisfaction with demographic data

	Satisfaction		Test of sig.	*p*
	Mean ± SD.	Median		
Gender				
Male	4.48 ± 0.37	4.55	U= 386.0	0.398
Female	4.52 ± 0.58	4.70		
Age				
< 22	4.38 ± 0.87	4.70	H= 0.682	0.711
22–24	4.57 ± 0.34	4.60		
≥ 25	4.36 ± 0.52	4.20		
University				
PUA	4.53 ± 0.66	4.70	H = 2.882	0.410
6 October	4.47 ± 0.38	4.50		
Alexandria	4.48 ± 0.32	4.60		
AAST	4.55 ± 0.07	4.55		
University type				
Public	4.48 ± 0.32	4.60	U = 442.0	0.184
Private	4.52 ± 0.62	4.70		
GPA				
< 2	4.50 ± 0.0	4.50	H= 1.027	0.792
2–2.49	4.58 ± 0.35	4.70		
2.5–3	4.55 ± 0.38	4.70		
> 3	4.48 ± 0.64	4.60		
University type				
Undergraduates	4.53 ± 0.55	4.70	U = 87.0	0.076
Postgraduates	4.26 ± 0.38	4.20		

U: Mann Whitney test H: H for Kruskal Wallis test

PUA: Pharos University in Alexandria

AAST: Arab academy for science and technology

GPA: Grade Point Average

p: *p* value for comparison between the studied categories

*: Statistically significant at *p* ≤ 0.05

**Table 9:** Correlation between overall knowledge and self-efficacy and self-esteem (*n* = 72)

		Overall knowledge	
		**Pre**	**Post**
Self-efficacy	**r**_**s**_	0.344^*^	0.111
	**p**	0.003^*^	0.354
Self-esteem	**r**_**s**_	0.173	-0.105
	**p**	0.147	0.381
Overall	**r**_**s**_	0.370^*^	0.036
	**p**	0.001^*^	0.766

r_s_: Spearman coefficient

*: Statistically significant at *p* ≤ 0.05

**Table 10:** Correlation between satisfaction with overall knowledge and self-efficacy and self-esteem (*n* = 72)

		Satisfaction	
		**r**_**s**_	**p**
Knowledge	**Pre**	-0.112	0.348
	**Post**	0.037	0.756
Self-efficacy	**Pre**	0.050	0.677
	**Post**	0.319^*^	0.006^*^
Self-esteem	**Pre**	-0.162	0.173
	**Post**	0.034	0.778
Overall	**Pre**	-0.028	0.813
	**Post**	0.215	0.069

r_s_: Spearman coefficient

*: Statistically significant at *p* ≤ 0.05

## Discussion

This is one of the few interventional studies demonstrating a detailed training program for pharmacy students in critical care, and it is the first to quantitatively assess self-efficacy and self-esteem and compare knowledge before and after the real-life critical care training course. The hospital administration was sufficiently supportive of undertaking the training program. Our findings demonstrated that the offered training was easy to implement and positively impacted pharmacy students’ knowledge, self-efficacy, and self-esteem regarding critical care patient management. Likewise, a previous online module about providing healthcare for individuals with diabetes intending to fast in Ramadan positively increased the self-efficacy and knowledge of pharmacy students and pharmacists [44]. Another study evaluated the effect of a critical course on knowledge gain in Rwanda and found that the course increased healthcare providers’ knowledge and confidence in various critical care topics [45].

Our training program offered the students the opportunity to practice the integrated learning materials, which refined their communication and bolstered their confidence, as demonstrated in the post-training self-efficacy, overall self-efficacy and self-esteem, and course satisfaction surveys, in line with previous studies where experiential learning improved the confidence of the majority of participants [18, 44]. On the contrary, a previous study found no difference in students’ self-efficacy perceptions after applying an experiential learning simulation program [37]. According to the WHO, health education is not merely knowledge but also acquiring the motivation, skill-building, and increasing the confidence (self-efficacy) necessary to improve health during practice [46]. Within experiential learning, opportunities exist for trainees to build their self-esteem and self-efficacy through engagement in hands-on, real-life activities [17]. Pharmacy students’ self-efficacy and self-esteem scores correlate to their ability to successfully overcome many challenges and have the confidence to take on various tasks effectively, including those in the critical care unit.

Being confident and motivated are the personal characteristics most required of future critical care pharmacists [5]. A multi-disciplinary community of practice exposed students to the importance of mutual trust and respect among different members of the healthcare team. This could have increased their consultation and problem-solving skills, reinforced the way they approach patients, and contributed to their acquisition of confidence by both working independently and having feedback from their preceptors if there was something they were unsure about [29, 47]. Participation in clinical rotations and having direct patient-facing experience can increase pharmacy students’ confidence in communication and contribute to a better understanding of the multidisciplinary healthcare working team [48, 49].

The pre-training findings reported here indicate that pharmacy students had low self-efficacy and self-esteem scores and lacked knowledge of key topics related to pain and agitation, phenytoin, and EBM. This was expected since pharmacy students were usually not introduced to experiential critical care learning earlier [1, 5]. A previous study carried in Saudi Arabia found that the majority of pharmacists had inadequate knowledge of pain and a negative attitude towards it management [50]. Contrary to our findings, a previous study found that students in US and Japan understood some common EBM terms [51]. However, the training course significantly advanced the trainees’ knowledge regarding critical care. This might have been improved by the use of multiple learning styles in the training course, which provided participants with a unique experience of critical care learning [52, 53].

The provision of training programs was previously recommended to optimize patient outcomes [54]. The implications for critical care pharmacy training explored herein aim to advance pharmacy education in low-to-middle-income countries (LMICs). When comparing public university students with private ones, we found that baseline self-efficacy and overall self-efficacy and self-esteem were higher in public university students compared to those in private universities. A previous study carried out on medical students [55] revealed that the self-esteem of healthcare students in private schools was significantly higher than that of those who attended public schools, which is contrary to our findings. Students with a GPA of more than 3 had significantly higher self-efficacy and overall self-efficacy and self-esteem scores. However, a study on Malaysian pharmacy students found no significant correlation between self-esteem level and GPA [56]. Self-efficacy and self-esteem did not significantly correlate with age. However, a previous study reported that the self-confidence of cardiology critical care physician trainees increased with age [31]. Likewise, although self-efficacy, self-esteem, and overall self-efficacy and self-esteem increased in males more than females after completion of the training program, the difference between the genders was not statistically significant. On the other hand, a self-confidence gender gap post-training in favor of males was reported previously [31, 57, 58].

Analyzing the responses from the self-satisfaction questionnaire filled out by students demonstrates a high satisfaction score with the critical care training course. Most students admitted that they enjoyed the course. Moreover, the course enhanced their knowledge of critical care and gave them the skills they wanted, and the learning experience was even better than expected. Medical students reported similar results of course satisfaction after adopting simulation-based teaching methods in anesthesia and intensive care [59]. High satisfaction scores after practical face-to-face training courses might be interpreted as a positive experience [60, 61]. Course satisfaction significantly positively correlated with the reported self-efficacy post-training.

Our findings not only confirm but also add to studies on training for pharmacy students by providing insights into how critical care experiential learning positively impacts pharmacy learners’ development. The main strength of this training course was using smaller groups of trainees to allow for better experiential learning [62].

Our results can be applied to other LMICs, which face constraints in resources and training. Training programs tailored to national needs of pharmacy students can be developed in other countries to bolster the capabilities of pharmacy students, especially in critical care, as demonstrated in this work.

## Limitations

This study has several limitations. First, we recruited a small number of participants, which might have increased the chance of type 2 error occurrence, underestimated the impact of the training program, and limited the generalizability of our findings. A larger sample of pharmacy students could have provided more representative data. Second, the follow-up time duration was only 10 days, which is a short period for students to gain most of the information they need for critical care practice; however, previous research reported an even shorter duration of critical care provision for pharmacy students [63]. A long-duration study may be needed to capture gains in learning, and how much the knowledge is retained and applied in the future.

Third, the practical competencies of the students, such as physical assessment skills, counseling skills, direct care provision skills, and their abilities to recommend valid interventions, were not assessed. The assessed parameters, such as self-efficacy, self-esteem, and knowledge, may not necessarily correlate with an improvement in technical skills and clinical performance. The study relied on a subjective self-assessment of self-efficacy and self-esteem rather than an objective assessment of competence. We can’t rule out the possibility that trainees were either overestimating or underestimating their actual capabilities.

## Recommendations

In light of our promising results, we suggest the replication of this study with a larger number of students in multi-centers to reshape the clinical competence of pharmacy students in critical care and increase their active involvement. Opinion leaders and stakeholders can adapt both the training programs and the assessment methods to become more objective and standardized. We acknowledge the necessity of designing a standardized critical care experiential learning program, awaited by many pharmacy students, to ensure the achievement of the minimum required skills and knowledge sets.

## Conclusion

Early findings from employing this elective critical care training given to pharmacy students revealed a positive impact on participants’ knowledge, confidence, self-efficacy, and self-esteem levels. The trainees valued the authentic patient-facing learning experience they received and were satisfied by the end of the critical care training program. The innovative training course was well-received by trainees and was effective. We recommend expanding the scalability of this training model and adapting it to similar LMICs globally. By doing so, we hope that our findings could serve as a framework for improving critical care education and reforming the pharmacy curricula. This can spark an interest in pharmacy students in critical care, and prepare them to play their role in critical care units with confidence.

## Electronic supplementary material

Below is the link to the electronic supplementary material.

Supplementary Material 1

## Data Availability

Availability of Data and Materials: Data are available from corresponding authors ( AB.K and HAAM ) upon reasonable request.

## Abbreviations

AAST: Arab Academy for Science and Technology
CKD: Chronic kidney disease
GPA: Grade Point Average
IQR: Inter-quartile range
LMICs: Low to middle-income countries
PUA: Pharos University in Alexandria
WHO: World Health Organization
