# Supplementary material for: Assessing the impact of critical care training on pharmacy students in Egypt: a pre-post study
Source: BMC Med Educ. 2024 Dec 20;24:1504. doi: 10.1186/s12909-024-06427-6 (PMC11661055; doi:10.1186/s12909-024-06427-6)
Supplement: Supplementary file 1 — Supplementary Material 1 [file 12909_2024_6427_MOESM1_ESM.docx]

**Table (1S):** **Pre/post-test for pain and agitation**

| Pre/post test for pain and agitation | | |
| --- | --- | --- |
| No | Question | Answers (correct answer is in bold) |
|  | One test of the following used to assess the pain in intubated patient ? a One test of the following used to assess the pain in intubated patient ? | a)RAMSY  **b)C-POT**  c)CAM-ICU  d)GCS |
|  | What is the first line analgesics used in treatment in critically ill patient (Significant pain) | **a)Opioid**  b)NSAIDS  c)Paracetamol  d)Aspirin |
|  | What is the first line sedative for critically ill patient? | **a)Dexmedetomidine (Precedex)**  b)Midazolam  c)Ketamine  d)Fentanyl |
|  | Which of the following sedative can increase the risk of delerium ? | a)Propofol  b)Dexmedetomidine (Precedex)  **c)Midazolam**  d)Haloperidol |
|  | All are causes of agitation in ICU except ? | a)Hypoglycemia  b)Pain  **c)Seizures**  d)Hypoxia |

**Table (2S):** **Pre/post-test for delirium**

| Pre/post-test for delirium | | |
| --- | --- | --- |
| No | Question | Answers (correct answer is in bold) |
|  | Types of delirium as/are …. | 1. Hypoactive 2. Hyperactive 3. Mixed 4. **All of the above** |
|  | …….. is used for the assessment for delirium | 1. RAMSY 2. **CAMICU** 3. BPS 4. C-POT |
|  | Used for prevention and treatment of delirium in icu | 1. Immobility 2. **Using the eye glasses** 3. Sleeping 4 hrs per day 4. Benzodiazepine use for treatment of agitation |
|  | Used for the treatment of delirium | 1. **Haloperidol** 2. Dexmedetomedine 3. Nalbuphin 4. Fentanyl |

**Table (3S):** **Pre/post-test for stroke**

| Pre/post-test for stroke | | |
| --- | --- | --- |
| No | Question | Answers (correct answer is in bold) |
|  | What is another name for a stroke? | 1. Heart attack 2. **Brain attack** 3. Myocardial infarction 4. None of the above |
|  | An ischemic stroke occurs when a blood clot blocks a blood vessel to the brain. | 1. **True** 2. False |
|  | Which of these is a symptom of stroke? | 1. Sudden confusion 2. Sudden weakness in an arm or leg 3. Sudden severe headache with no cause 4. Sudden trouble seeing 5. **All of the above** |
|  | Which of these factors plays the biggest role in increasing the risk for stroke in younger adults? | 1. Overweight 2. Little or no exercise 3. **High blood pressure** 4. Smoking |
|  | If a person has an ischemic stroke, how quickly should the person be treated to minimize long-term problems? | 1. Within 30 minutes 2. Within 1 hour 3. Within 2 hours 4. **Within 3 hours** |
|  | Which type of medicine is given to help prevent a stroke? | 1. Medicine to prevent clots from forming 2. Blood-thinner medicine 3. Clot-busting medicine 4. **A and B** 5. All of the above |
|  | Which of these may be a long-term problem after a stroke? | 1. Paralysis or weakness on one side of the body 2. Problems with thinking or memory 3. Problems with language 4. Pain in the hands and feet 5. **All of the above** |

**Table (4S):** **Pre/post-test for heart failure**

| Pre/post-test for heart failure | | |
| --- | --- | --- |
| No | Question | Answers (correct answer is in bold) |
|  | What is a systolic heart failure? | 1. A condition in which the heart stops beating. 2. **A condition in which the heart cannot pump enough blood to meet the needs.** 3. A heart attack. 4. A condition in which the patient experiences chest pain |
|  | Congestive heart failure and heart failure are the same? | 1. True 2. **False** |
|  | What are common signs and symptoms of heart failure? | 1. Shortness of breath or trouble breathing 2. Fatigue 3. Swelling 4. **All of the above** |
|  | A patient can have heart failure without being aware of it. | 1. **True** 2. False |
|  | Which patient is the best candidate for initiation of ivabradine? | 1. 55-year-old man with NYHA functional class I HF (LVEF of 60%) and heart rate 75 beats/ minute taking lisinopril 20 mg daily for HTN. 2. 45-year-old man with NYHA functional class II HF (LVEF of 35%) and heart rate 62 beats/minute taking lisinopril 40 mg daily, metoprolol succinate 150 mg daily, and spironolactone 25 mg daily. 3. **65-year-old woman with NYHA functional class III HF (LVEF of 20%) with heart rate 78 beats/minute taking lisinopril 20 mg daily, eplerenone 50 mg daily, and carvedilol 25 mg twice daily.** 4. 85-year-old man with NYHA function class IV HF (LVEF of 10%) with heart rate 110 beats/minute receiving outpatient dobutamine therapy and awaiting transplantation. |

**Table (5S):** **Pre/post-test for atrial fibrillation**

| Pre/post-test for atrial fibrillation | | |
| --- | --- | --- |
| No | Question | Answers (correct answer is in bold) |
|  | What is the initial investigative study to confirm Atrial fibrillation? | 1. Echo 2. **ECG** 3. CT scan 4. EEG |
|  | CHA2DS2-VASc score is a validated scoring system for assessing the risk of stroke in? | 1. Valvular Atrial Fibrillation 2. **Nonvalvular Atrial Fibrillation** |
|  | Selection of an anticoagulation agent should be based on stroke risk in which of the following? | 1. Paroxysmal AF 2. Persistent AF 3. Permanent AF 4. **All of the above** |
|  | Which of the following can be termed Persistent atrial fibrillation? | 1. Acute onset of atrial fibrillation with spontaneous termination 2 days later. 2. Atrial fibrillation that has been continuously present for 4 years. 3. **AF that is present for 10 days and required electrical cardioversion.** 4. AF occurring an average of 5 times weekly eventually requiring antiarrhythmic drug therapy to maintain sinus rhythm. |
|  | Surgical options for rate control consist of AV nodal ablation and implantation of a permeant ventricular pacemaker? | 1. **True** 2. False |

**Table (6S):** **Pre/post-test for sepsis**

| Pre/post-test for sepsis | | |
| --- | --- | --- |
| No | Question | Answers (correct answer is in bold) |
|  | Most common infection that lead to sepsis : | 1. **Lung infection** 2. Urinary tract infection 3. Gut infection 4. Skin infection |
|  | Patient in a septic shock , …………… will be used | 1. Narrow spectrum antibiotics 2. **Broad spectrum antibiotics** 3. Antifungal |
|  | A crystalloid bolus of 30 ml/Kg is recommended within ….. hrs | 1. **3** 2. 6 3. 2 |
|  | A rise in lactate during sepsis can be due to | 1. Liver failure 2. Tissue hypoxia 3. Some medications such as (epinephrine, beta-2 agonists) 4. **All of the above** |
|  | SOFA is an objective scoring system | 1. **Organ dysfunction** 2. For de-escalation of antibiotic 3. For antibiotic resistance |

**Table (7S):** **Pre/post-test for pneumonia**

| Pre/post-test for pneumonia | | |
| --- | --- | --- |
| No | Question | Answers (correct answer is in bold) |
|  | If MRSA is suspected in Patients with S. aureus infection, what is the most appropriate antibiotic in their case? | 1. Tetracycline. 2. Erythromycin. 3. Clindamycin. 4. **Vancomycin** |
|  | Typical bacterial pneumonia is caused by: | 1. Mycoplasmal pneumonia. 2. Legionnaires pneumonia. 3. Pneumocystis carinii. 4. **Staphylococcus aureus.** |
|  | A 42 shepherd came to the primary care with chest pain and cough. What type of pneumonia does he have? | 1. **CAP.** 2. HAP. 3. VAP |
|  | Pneumonia that caused following passage of food particles ,drink into the lung is called: | a) CAP  b) **Aspiration pneumonia**  c) Atypical pneumonia  d) None of the above |
|  | Which of these cause atypical pneumonia: | a)Pneumococcus  b)Influenza  c)M**ycoplasma**  d)Viruses |

**Table (8S):** **Pre/post-test for CKD**

| Pre/post-test for CKD | | |
| --- | --- | --- |
| No | Question | Answers (correct answer is in bold) |
|  | …………..slow disease progression and safely control blood pressure | 1. **ACEI,ARBs** 2. Diuretics 3. CCBs |
|  | We must treat iron deficient anemia before giving ESA | 1. **True** 2. False |
|  | "An 82-year-old African American man with history of well controlled hypertension is referred for further evaluation of Dover the past 6 months, his appetite has been poor and his weight has decreased by 5 kg. He appears chronically ill with decreased muscle mass on physical examination. his weight is 5.4 kg with a body mass index of 17.5 kg/㎡. Laboratory studies shown Ser level of 2.6 mg/dl. Which one of the following methods will accurately estimate this patient's renal function? | a)Modification in Diet in Renal Disease (MDRD) formula.  b)Creatinine clearance.  c)Chronic Kidney Discase Epidemiology Collaboration (CKD-EPI) creatinine formula.  **d)CKD-EPI cystatin C formula."** |
|  | "Most preferred agents to bind intestinal phosphate: | **a)** **Calcium carbonate and calcium acetate**  b)Aluminum hydroxide and calcium carbonate  c)Calcium citrate and calcium acetate" |
|  | "salt intake to (less than.........)per day of sodium in CKD to avoid fluid overload | **a)2g**  b)3g  c)1g" |

**Table (9S):** **Pre/post-test for electrolyte disturbance**

| Pre/post-test for electrolyte disturbance | | |
| --- | --- | --- |
| No | Question | Answers (correct answer is in bold) |
|  | What is the maximum infusion rate for IV potassium chloride in a patient has a peripheral line? | 1. **10 meq/hr** 2. 20 meq/hr 3. 30 meq/hr 4. 40meq/hr |
|  | Calcium gluconate is used in the antihyperkalemic regimen because it has antihyperkalemic effect? | a)Yes b) **No** |
|  | Mineral water can be used in the treatment of Hypernatremia? | a) Yes b) **No** |
|  | Usually we use ionized calcium level to detect any problem in the calcium level ? | a)**Yes**  b) No |
|  | In severe hyperphospatemia, we use which agent to lower phosphate level ? | 1. Hypertonic saline (3%) 2. **Renagel(sevelamer)** 3. Mannitol 4. Tridil |

**Table (10S):** **Pre/post-test for phenytoin**

| Pre/post-test for phenytoin | | |
| --- | --- | --- |
| No | Question | Answers (correct answer is in bold) |
|  | What does TDM stand for ? …………… | 1. **Therapeutic drug monitoring** 2. Team Decision Making meeting 3. Test Data Management 4. Time division multiplexing |
|  | Phenytoin is an ……. | 1. **Enzyme inducer** 2. Enzyme inhibitor |
|  | The active form of phenytoin is the …… | 1. **Free phenytoin** 2. Bound phenytoin 3. Both |
|  | One of the following is a risk factor for phenytoin toxicity | 1. Hypokalemia 2. **Hypoalbuminemia** 3. Hypocalcemia |
|  | Phenytoin kinetics follows …….. | a)1^st^ order kinetics b) **M-M kinetics** c) linear kinetics |

**Table (11S):** **Pre/post-test for evidence-based medicine**

| Pre/post-test for evidence-based medicine | | |
| --- | --- | --- |
| No | Question | Answers (correct answer is in bold) |
|  | All the following cannot be taken via NGT/OGT except ……….. | a) Aspirin protect (enteric coated tab)  b) Isoptin XR  c) **Norvasc**  d) Depakin chrono |
|  | …………….. is used as a DVT prophylaxis | 1. **Clexane** 2. Aspirin 3. Plavix 4. Brilique |
|  | Who is eligible for SUP ( stress ulcer prophylaxis) ………. | 1. Pt with pneumonia 2. **Pt on vent for more than or equal to 48hrs** 3. Pt with INR =1 4. Pt with pulmonary edema (not intubated) |
|  | All of the following can be used as a trusted website or APP for searching for the dose of meronam in pt with HAP (Hospital acquired pneumonia) except ………… | 1. Lexicomp 2. EMC 3. **ESC** 4. Micromedex |
|  | All the following are trusted reference for choosing the empirical antibiotic in pt with cellulitis except ………… | 1. IDSA 2. Sanford 3. Johnhopkins 4. **Lexicomp** |
|  | Drug-drug interaction can be checked in ………. | 1. Globalrph 2. **Drugs.com** 3. Sider.com 4. Labtest online |
|  | Monitor in icu contain the following except………. | 1. Heart rate 2. Respiratory rate 3. **CO2 saturation** 4. Blood pressure |

**Table (12S): Self-efficacy and self-esteem**

| Self-efficacy and self-esteem pre- and post-training | | | | | | |
| --- | --- | --- | --- | --- | --- | --- |
| No | Statement | Answers | | | | |
|  |  | Strongly disagree | Disagree | Can’t dictate | agree | Strongly agree |
|  | I will be able to achieve most of the goals that I have set for myself |  |  |  |  |  |
|  | When facing difficult tasks, I am certain that I will accomplish them. |  |  |  |  |  |
|  | In general, I think that I can obtain outcomes that are important to me. |  |  |  |  |  |
|  | I believe I can succeed at most any endeavor to which I set my mind. |  |  |  |  |  |
|  | I will be able to successfully overcome many challenges. |  |  |  |  |  |
|  | I am confident that I can perform effectively on many different tasks. |  |  |  |  |  |
|  | Compared to other people, I can do most tasks very well. |  |  |  |  |  |
|  | Even when things are tough, I can perform quite well. |  |  |  |  |  |
|  | I feel that I am a person of worth, at least on an equal plane with others |  |  |  |  |  |
|  | I feel that I have a number of good qualities |  |  |  |  |  |
|  | All in all, I am inclined to feel that I am a failure |  |  |  |  |  |
|  | I am able to do things as well as most people. |  |  |  |  |  |
|  | I feel that I do not have much to be proud of. |  |  |  |  |  |
|  | I take a positive attitude toward myself |  |  |  |  |  |
|  | On the whole, I am satisfied with myself. |  |  |  |  |  |
|  | I wish I could have more respect for myself. |  |  |  |  |  |
|  | I certainly feel useless at times |  |  |  |  |  |
|  | At times I feel I am not good at all |  |  |  |  |  |

**Table (13S): Course-satisfaction questionnaire**

| No | Statement | Strongly disagree | Disagree | Can’t decide | Agree | Strongly agree |
| --- | --- | --- | --- | --- | --- | --- |
| 1 | Overall, I enjoyed the course |  |  |  |  |  |
| 2 | Overall, the course improved my knowledge of critical care |  |  |  |  |  |
| 3 | Overall, the course gave me the experience/skills I wanted or needed |  |  |  |  |  |
| 4 | Overall, the course met my learning needs |  |  |  |  |  |
| 5 | Overall, the learning experience was better than expected |  |  |  |  |  |
| 6 | Overall, the content of the course was easy to follow |  |  |  |  |  |
| 7 | Overall, I am satisfied with the pace of the course |  |  |  |  |  |
| 8 | Overall, I am satisfied with the way the course was delivered |  |  |  |  |  |
| 9 | Overall, the skills and knowledge acquired during the course will help me in my job |  |  |  |  |  |
| 10 | Overall, the course is very useful for pharmacy students |  |  |  |  |  |

**Table (14S): Comparison between pre- and post-training according to knowledge scores
(n = 72)**

|  | Pre | Post | p |
| --- | --- | --- | --- |
| Pain and agitation |  |  |  |
| Total Score | **(0–5)** |  | <0.001^*^ |
| Min. – Max | 0.0 – 5.0 | 3.0 – 5.0 |  |
| Median (IQR) | 1.0 (1.0 – 3.0) | 5.0 (5.0–5.0) |  |
| Delirium |  |  |  |
| Total Score | **(0–4)** |  | <0.001^*^ |
| Min. – Max | 0.0 – 4.0 | 2.0 – 4.0 |  |
| Median (IQR) | 2.0 (1.0–2.0) | 4.0 (4.0–4.0) |  |
| Stroke |  |  |  |
| Total Score | **(0–7)** |  | <0.001^*^ |
| Min. – Max | 2.0 – 7.0 | 5.0 – 7.0 |  |
| Median (IQR) | 5.0 (5.0–6.0) | 7.0 (7.0–7.0) |  |

IQR: **Inter quartile range** SD: **Standard deviation**

**Z: Wilcoxon signed ranks test**

p: p value for comparing between **pre and post**

*: Statistically significant at p ≤ 0.05

**Table (15S): Comparison between pre- and post- training according to knowledge scores
(n = 72) "continue"**

|  | Pre | Post | p |
| --- | --- | --- | --- |
| Heart failure |  |  |  |
| Total Score | **(0–5)** |  | <0.001^*^ |
| Min. – Max | 2.0 – 5.0 | 4.0 – 5.0 |  |
| Median (IQR) | 4.0 (3.0–5.0) | 5.0 (5.0–5.0) |  |
| Atrial fibrillation |  |  |  |
| Total Score | **(0–5)** |  | <0.001^*^ |
| Min. – Max | 0.0 – 5.0 | 4.0 – 5.0 |  |
| Median (IQR) | 3.0 (2.0–4.0) | 5.0 (5.0–5.0) |  |
| Sepsis |  |  |  |
| Total Score | **(0–5)** |  | <0.001^*^ |
| Min. – Max | 0.0 – 5.0 | 3.0 – 5.0 |  |
| Median (IQR) | 3.0 (2.0–4.0) | 5.0 (5.0–5.0) |  |

IQR: **Inter quartile range** SD: **Standard deviation**

**Z: Wilcoxon signed ranks test**

p: p value for comparing between **pre and post** *: Statistically significant at p ≤ 0.05

**Table (16S): Comparison between pre- and post- training according to knowledge scores
(n = 72) "continue"**

|  | Pre | Post | p |
| --- | --- | --- | --- |
| Pneumonia |  |  |  |
| Total Score | **(0–5)** |  | <0.001^*^ |
| Min. – Max | 0.0 – 5.0 | 4.0 – 5.0 |  |
| Median (IQR) | 4.0 (3.0–4.0) | 5.0 (5.0–5.0) |  |
| CKD |  |  |  |
| Total Score | **(0–5)** |  | <0.001^*^ |
| Min. – Max | 0.0 – 5.0 | 3.0 – 5.0 |  |
| Median (IQR) | 3.0 (2.0–4.0) | 5.0 (5.0–5.0) |  |
| Electrolyte disturbance |  |  |  |
| Total Score | **(0–5)** |  | <0.001^*^ |
| Min. – Max | 0.0 – 5.0 | 3.0 – 5.0 |  |
| Median (IQR) | 3.0 (2.0–3.0) | 5.0 (5.0–5.0) |  |

IQR: **Inter quartile range**

**Z: Wilcoxon signed ranks test**

**CKD : Chronic kidney disease**

p: p value for comparing between **pre and post**

*: Statistically significant at p ≤ 0.05

**Table (17S): Comparison between pre and post according to knowledge scores
(n = 72) "continue"**

|  | Pre | Post | p |
| --- | --- | --- | --- |
| Phenytoin |  |  |  |
| Total Score | **(0–5)** |  | <0.001^*^ |
| Min. – Max | 0.0 – 5.0 | 4.0 – 5.0 |  |
| Median (IQR) | 2.0 (1.0–3.0) | 5.0 (5.0–5.0) |  |
| Evidence based medicine |  |  |  |
| Total Score | **(0–7)** |  | <0.001^*^ |
| Min. – Max | 0.0 – 6.0 | 4.0 – 7.0 |  |
| Median (IQR) | 3.0 (2.0–4.0) | 7.0 (7.0–7.0) |  |
| Overall knowledge |  |  |  |
| Total Score | **(0–58)** |  | <0.001^*^ |
| Min. – Max | 21.0 – 45.0 | 53.0 – 58.0 |  |
| Median (IQR) | 31.0 (29.0–35.50) | 57.0 (56.0–58.0) |  |

IQR: **Inter quartile range**

**Z: Wilcoxon signed ranks test**

CKD: chronic kidney disease

p: p value for comparing between **pre and post**

*: Statistically significant at p ≤ 0.05
